# Identification and validation of tissue-based gene biomarkers for acute intestinal graft-versus-host disease(AIGVHD)

**DOI:** 10.3389/fimmu.2025.1574904

**Published:** 2025-05-13

**Authors:** Hong Chen, Liyu Fu, Liying Liu, Yunyan He

**Affiliations:** ^1^ Department of Pediatrics, The First Affiliated Hospital of Guangxi Medical University, Nanning, China; ^2^ Graduate School, Guangxi Medical University, Nanning, China; ^3^ The Key Laboratory of Children’s Disease Research in Guangxi’s Colleges and Universities, Education Department of Guangxi Zhuang Autonomous Region, Nanning, China; ^4^ The first afflicted hospital of Guangxi medical university/Difficult and Criticaillness Center, Pediatric Clinical Medical Research Center of Guangxi, Nanning, China; ^5^ NHC Key Laboratory of Thalassemia Medicine, Guangxi Medical University, Nanning, China; ^6^ Guangxi Key Laboratory of Thalassemia Research, Guangxi Medical University, Nanning, China

**Keywords:** aGVHD, Allo HSCT, gene biomarkers, immune cell infiltration, machine learning, aGVHD mouse model

## Abstract

**Background:**

Acute intestinal graft-versus-host disease (AIGVHD) is a common complication of allogeneic hematopoietic stem cell transplantation (allo HSCT) with a high mortality rate. The primary aim of the present study is to identify tissue-based gene biomarkers pertinent to AIGVHD, thereby facilitating early diagnosis and exploration of potential therapeutic targets.

**Method:**

The dataset was obtained from the GEO database. DEGs were identified, followed by GO and KEGG pathways analysis for the common DEGs. PPI networks and WGCNA analysis were used to identify essential genes, and correlations between critical genes and immune cell infiltration were also examined. The diagnostic efficacy of these essential genes was evaluated using ROC curves, leading to the development of 11 machine learning models based on this gene set. Furthermore, we established a mouse model of aGVHD, which was identified by clinical score, pathological analysis, flow cytometry detection of implantation rate, and immunohistochemical detection of CD4 expression. Finally, we measured the mRNA expression levels of the key genes in the mice’s intestinal tissue using real-time PCR.

**Result:**

DEGs showed a marked enrichment in immune and inflammatory response pathways. Our analysis identified three key genes, FCGR3A, SERPING1, and IFITM3, which were positively associated with M1 macrophage and neutrophil infiltration. Subsequently, we developed machine learning models utilizing these three genes and found that the RF model exhibited a robust predictive capacity for AIGVHD occurrence, achieving an AUC of 0.9802 (95% CI: 0.966–0.9945). An aGVHD mouse model was also successfully created, and we discovered that the aGVHD group’s mRNA expression levels of three key genes were noticeably higher than the control group’s.

**Conclusion:**

In this study, we identified FCGR3A, SERPING1, and IFITM3 as tissue-based gene biomarkers for AIGVHD, highlighting their diagnostic efficacy. Furthermore, we confirmed the association of these genes with AIGVHD through investigations conducted in aGVHD mouse models.

## 1 Introduction

Allogeneic hematopoietic stem cell transplantation (allo-HSCT) represents a pivotal therapeutic strategy for a variety of hematological disorders and primary immunodeficiencies (1–3). Recent trends indicate a marked increase in the global incidence of allo-HSCT procedures conducted annually (4, 5). Nonetheless, acute graft-versus-host disease (aGVHD) remains a prevalent and severe complication associated with allo-HSCT, contributing to significant morbidity and mortality (6–9). According to the National Institutes of Health, aGVHD occurring within the first 100 days post-transplantation is classified as classical aGVHD, whereas manifestations arising beyond this timeframe are categorized as delayed, recurrent, or persistent aGVHD (10, 11). Among the various organs affected by aGVHD, the gastrointestinal tract ranks as the second most commonly involved site, with gastrointestinal manifestations significantly elevating the risk of mortality in affected patients (12–14).

The diagnosis of acute intestinal graft-versus-host disease (AIGVHD) currently relies on the combination of clinical symptomatology and invasive tissue biopsy. Notably, patients with AIGVHD may exhibit atypical clinical presentations and lack definitive intestinal pathological changes in the initial phases of the disease. Consequently, it is imperative to identify diagnostic biomarkers that can effectively stratify high-risk patients at potential risk for developing AIGVHD. Recent investigations have focused extensively on potential biomarkers, with serum markers such as TNFR1, TIM3, ST2, REG3α, Elafin, and Amphiregulin along with alterations in intestinal microbiota, showing promise in both the diagnosis and prediction of AIGVHD (15–20). Furthermore, elevated levels of CD25 and CTLA-4 mRNA have been correlated with the manifestation, severity, and progression of AIGVHD (21). Despite these advancements, no gene-level biomarker has yet been validated as a reliable diagnostic tool for AIGVHD.

The pathogenesis of aGVHD involves the direct recognition of the recipient’s major histocompatibility complex (MHC) by donor T lymphocytes, which subsequently undergo clonal expansion. This process is mediated by effector T cells and pro-inflammatory cytokines, which inflict damage on the epithelial cells of the gastrointestinal, skin, and liver, ultimately leading to apoptosis and programmed necrosis. Additionally, other immune cell populations may contribute to disease pathology (6, 22–24), underscoring the necessity for further investigation into the immune infiltration associated with aGVHD.

This study aims to employ bioinformatics methodologies to discern potential gene biomarkers derived from intestinal tissue for the prediction of AIGVHD while concurrently assessing immune infiltration within the intestinal microenvironment. Additionally, we will develop machine learning models based on the identified genetic biomarkers. To further validate critical genes, we will establish a murine model of aGVHD, aiming to enhance our understanding of the pathogenesis of AIGVHD and uncover potential therapeutic targets.

## 2 Materials and methods

### 2.1 Data source

The gene expression dataset pertinent to AIGVHD originated in the Gene Expression Omnibus (GEO) database, a public functional genomics resource (https://www.ncbi.nlm.nih.gov/geo/). The following were the requirements for inclusion in these datasets: (1) utilization of high-throughput sequencing technologies; (2) a minimum sample size of five per group to ensure statistical rigor; and (3) total RNA extraction from human colon tissue. Ultimately, three series were selected for analysis: GSE134662, GSE168116, and GSE215068. Gene expression profiles along with clinical annotation data were subsequently downloaded for further evaluation. **Supplementary Table S1** details the datasets.

### 2.2 Screening for hub genes

Differentially expressed genes (DEGs) were identified between the GSE134662 and GSE168116 datasets using the GEO2R tool. DEGs were defined by an adjusted P value < 0.05 and |log2 FC| ≥1, comparing patients with AIGVHD to those without No_AIGVHD. Common DEGs shared between the two datasets were subsequently determined through the online VENN analysis tool, Jvenn. Gene ontology (GO) and Kyoto encyclopedia of genes and genomes (KEGG) pathway enrichment analyses were performed on the common DEGs utilizing the clusterProfiler package (version 4.5.0) (25). Protein-protein interaction (PPI) networks for the DEGs were constructed using the STRING database. For visualization purposes, the interaction data were imported into Cytoscape (version 3.10.1), and the CytoNCA plugin was employed to identify hub genes within the network.

### 2.3 The network analysis of weighted gene co-expression

In this study, we utilized the “WGCNA” R package for the analysis of gene co-expression networks (26, 27). Initially, the expression profiles from GSE134662 and GSE168116 were integrated, utilizing the “combat” function from the “sva” package. We selected a power of β=9, a scale-free R2 = 0.85, a cut height of 0.25, and a minimum module size of 150 as soft-thresholding parameters, thereby establishing an unsigned co-expression gene network. Subsequently, the adjacency matrix was transformed into a topological overlap matrix (TOM), and the dynamic cut tree method algorithm was employed to discern gene clustering modules. Candidate genes were identified by applying the thresholds of module membership (MM)≥0.8 and gene significance (GS) >0.4. The data analysis was executed using the HiOmics Cloud Platform (https://henbio.com/en/tools) (28).

### 2.4 Identify critical genes and assess diagnostic efficacy

To identify common critical genes, the candidate genes derived from the PPI networks and WGCNA were subjected to Venn analysis. Furthermore, to evaluate the predictive capacity of these critical genes, receiver operating characteristic (ROC) curve analysis was carried out on the test dataset. To further validate the predictive value of the identified critical genes, an additional validation dataset was acquired, and ROC curve analysis was subsequently performed.

### 2.5 Machine learning models

To evaluate the predictive power of the three critical genes, we trained eleven machine learning models aimed at forecasting the occurrence of AIGVHD. The algorithms employed included Decision Tree (DT), Random Forest (RF), Logistic Regression (LR), Naive Bayes (NB), Support Vector Machine (SVM), XGBoost, Linear Discriminant Analysis (LDA), Generalized Linear Model with Elastic Net Regularization (GLMNET), k-Nearest Neighbor (KNN), Quadratic Discriminant Analysis (QDA), and Binary Stacking. To optimize model parameters, five-fold cross-validation was performed. This operation is implemented in R using the “tidyverse (2.0.0)” and “mlr3verse (0.2.8)” packages.

### 2.6 Immune microenvironment in AIGVHD

Immunity and the incidence of AIGVHD are closely associated. We will then investigate the immune microenvironment of colon tissue in patients with AIGVHD. Assess the abundancer of 22 immune cell types that could infiltrate the intestinal of AIGVHD using the CIBERSOR algorithm. The immune infiltration between AIGVHD patients and control patients was then compared using the Wilcoxon rank sum test. Spearman correlation analysis was used to investigate the connection between important genes and immune cell infiltration.

### 2.7 Mice

Male BALB/c mice (H-2kd) were utilized as donors and were purchased from Guangdong Weitong Lihua Experimental Animal Technology (Guangdong, China). Female C57BL/6J mice (H-2kb) were procured as recipients from the Experimental Animal Center of Guangxi Medical University (Nanning, China). All mice were aged between 8 and 9 weeks and had a weight range of 17 to 22g. The mice were maintained in a specific pathogen-free (SPF) environment at the Experimental Animal Center of Guangxi Medical University. All animal studies were approved by the Animal Ethics Committee of Guangxi Medical University.

### 2.8 aGVHD model

Following a two-week period of adaptive feeding, C57BL/6J mice were randomly divided into two experimental groups: a normal group and an aGVHD group, with six mice in each cohort. During the week preceding transplantation, all mice received sterile water supplemented with 320 mg/L of gentamicin (Yuanye, Shanghai, China), a protocol that continued post-transplantation. Mice in the aGVHD group underwent intraperitoneal injections of 38 mg/kg/d of Busulfan (Yuanye, Shanghai, China) for four days (from -7 to -4 days) and 120 mg/kg/d of Cyclophosphamide (Yuanye, Shanghai, China) for two days (from -3 to -2 days). On the seventh day, the aGVHD group was administered 0.2 mL of a bone marrow and spleen cell suspension harvested from BALB/c mice via the tail vein, containing 1×10^8 bone marrow cells and 2×10^8 spleen cells. In contrast, the normal group received 0.2 mL of RPMI 1640 solution. The procedure is illustrated in **Supplementary Figure S1**.

For a period of 14 days post-transplantation, the weight, posture, mobility, fur condition, and skin integrity of the mice were closely monitored. Clinical symptoms of GVHD were evaluated using a standardized scoring system (29). On the fourteenth day following transplantation, the mice were euthanized, and tissue samples were collected. Spleen lymphocytes were isolated using Mouse Lymphocyte Isolation Solution (Solarbio, Beijing, China), and the implantation rate was assessed using flow cytometry (BD VersFACSTM). For cell staining, FITC-conjugated anti-mouse H2kb antibodies (BD, USA) and PE-conjugated anti-mouse H2kd antibodies (BD, USA) were employed. Skin, liver, and colon samples were collected for hematoxylin and eosin staining (HE), as well as pathological damage assessment using the Lerner score (30).

The occurrence and progression of aGVHD are significantly influenced by CD4+ T cells. In this research, the CD4 expression was measured using immunohistochemistry. The staining protocol employed antibodies specific to CD4 (Servicebio, Wuhan, China, dilution 1:500) alongside HRP-conjugated secondary antibodies (Servicebio, Wuhan, China, dilution 1:200). Micrographs of the stained tissue sections were acquired with an optical microscope (Zeiss, Axio Imager 2, Germany), capturing three randomly selected visual fields for each section. The integral optical density (IOD) values and the positive area within each visual field were quantified using Image J software (NIH, USA). The relative expression of CD4 was evaluated by computing the average optical density (AOD) of the positive regions (IOD/area).

### 2.9 RNA isolation and QRT-PCR experiment

On the fourteenth day post-transplantation, colon tissues were harvested from both the control and aGVHD groups. Total RNA was isolated using the RNAeasy animal RNA extraction kit (Beyotime, Shanghai, China) and subsequently reverse transcribed into cDNA utilizing the reverse transcription kit (Vazyme, Nanjing, China). PCR amplification was performed with the HS Universal qPCR Master Mix (ACE, Nanjing, China) in a 7500 PCR instrument (Applied Biosystems, USA). The primers were synthesized by Bioengineering (Shanghai) Co., Ltd., with their sequences detailed in **Table 1**. The PCR reaction mixture comprised 0.4 μL (10 µM) of each primer, 1 μL of DNA template, 10 μL of the master mix, and 8.2 μL of deionized water. Denaturation at 95°C for 30 seconds was the first step in the PCR protocol. This was followed by 40 cycles of denaturation at 95°C for 10 seconds and annealing/extension at 60°C for 15 seconds. The program concluded with a melting curve analysis, recording fluorescence at 95°C for 15 seconds, 60°C for 60 seconds, and 95°C for 15 seconds. The relative expression levels of the target genes were determined using the 2^−△△CT^ method.

**Table 1:** PCR primer sequences.

Gene	Forward primer	Reverse primer
FCGR3A	CCACACCAGGATGCCAACTA	CTGAAGCAATAGCCAGCCCATA
SERPING1	TACTTTGAAGGCCAAGGTGGG	AGTGGGGTTGAGAGCCTTTT
IFITM3	CTATGCCTACTCCGTGAAGTCTA	CAATGGTGATAACAACCATCAGG
RPL32(reference genes)	TTAAGCGAAACTGGCGGAAAC	TTGTTGCTCCCATAACCGATG

### 2.10 Statistical analysis

Statistical analyses were conducted using SPSS version 26.0 (IBM, USA). Measurement results are expressed as mean ± SEM. For comparisons between two groups with homogenous variance, the Student’s t-test was applied. In cases where the data exhibited unequal variance and non-normal distribution, the Mann-Whitney U test was utilized (p<0.05 was statistically significant).

## 3 Results

### 3.1 Screening for hub genes

**Figure 1:**
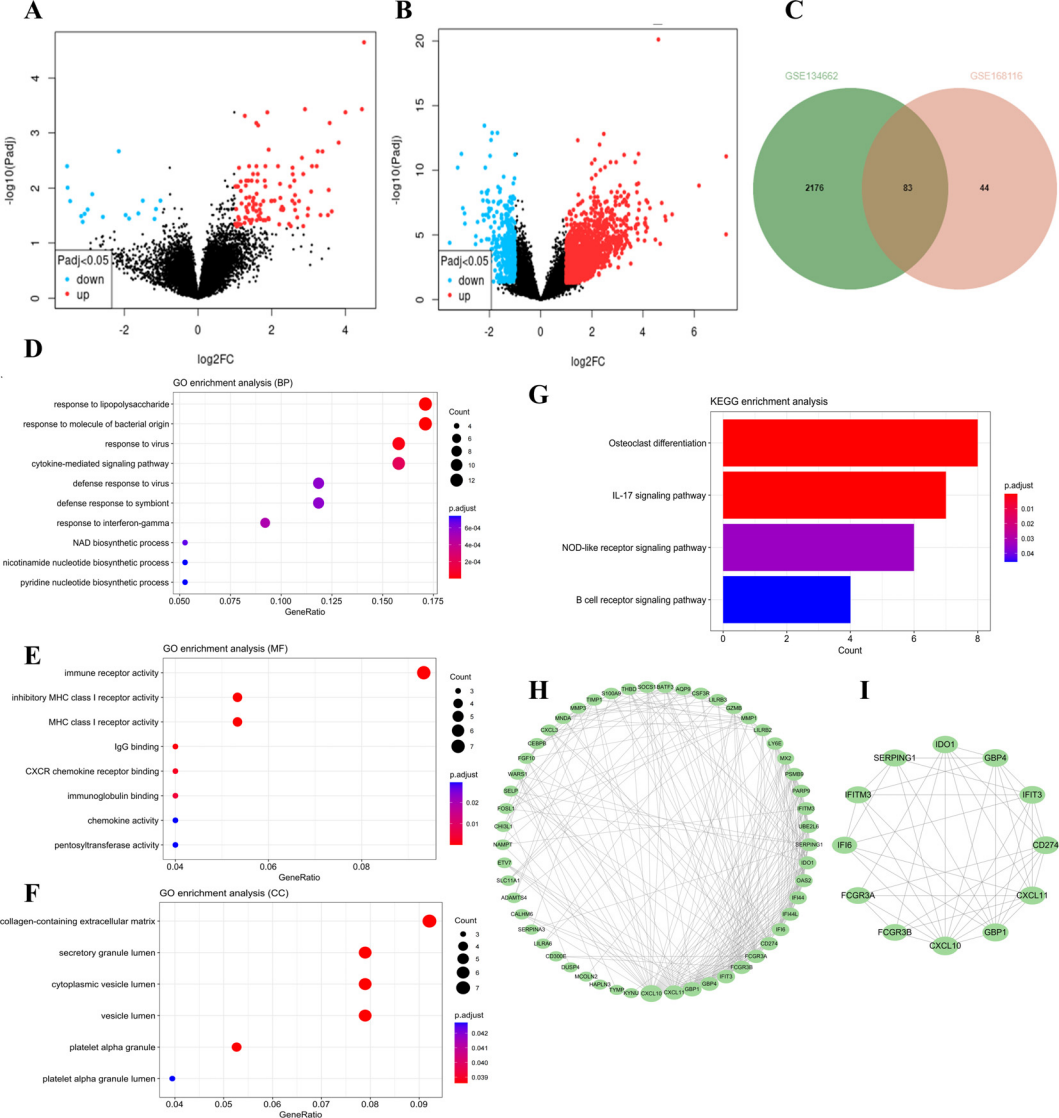
The differentially expressed genes in the data sets were screened, followed by functional enrichment analysis of the common DEGs. Candidate hub genes were determined through PPI network analysis. **(A)** The volcano plots of the GSE168116 dataset. **(B)** The volcano plots of the GSE134662 dataset. **(C)** The Venn diagram represents the common DEGs between two datasets. **(D-F)** GO functional analysis of the DEGs. **(G)** KEGG analysis of the DEGs. **(H)** PPI network analysis of the DEGs. **(I)** Identification of candidate hub genes utilizing the CytoNCA plug-in.

The “DESeq2” package was employed to identify DEGs within the GSE168116 and GSE134662 datasets, yielding 127 and 2259 DEGs, respectively (**Figures 1A, B**). Of these, 83 were common DEGs (**Figure 1C**, **Supplementary Table S2**). Subsequent GO functional enrichment analyses of the overlapping DEG revealed that immune and inflammatory responses were involved in the development of AIGVHD (**Figures 1D-F**, **Supplementary Tables S3**-**5**). These DEGs were primarily enriched in the IL-17 signaling pathway, NOD-like receptor signaling pathway, and B cell receptor signaling pathway, according to KEGG analysis (**Figure 1G**, **Supplementary Table S6**). To elucidate the interactions among the common DEGs, a PPI network was constructed utilizing the STRING database, with visualization facilitated by Cytoscape (version 3.10.1) (**Figure 1H**). Nodes with higher degrees have more edges and have a greater impact on the network, suggesting that they are involved in more biological processes. Through the application of the CytoNCA plug-in, twelve core genes—including FCGR3A, FCGR3B, IDO1, CXCL10, CD274, GBP1, GBP4, CXCL11, IFIT3, SERPING1, IFITM3, and IFI6—were delineated as candidate genes for further investigation (**Figure 1I**).

### 3.2 The WGCNA analysis

**Figure 2:**
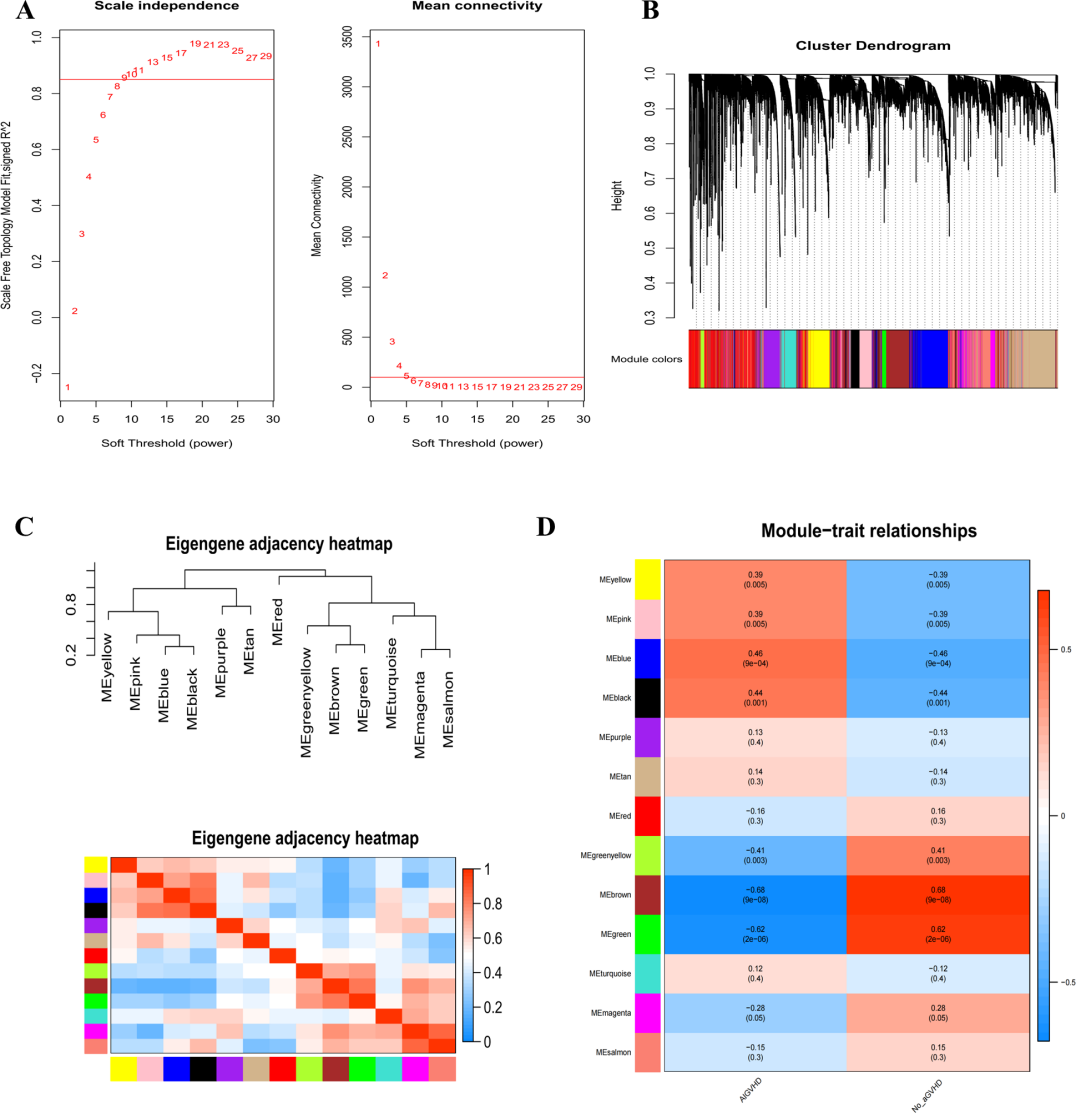
Use WGCNA analysis to identify modules associated with AIGVHD. **(A)** The mean connectivity and scale independence of eigengenes. **(B)** The gene clustering tree diagram uses clustering to find very similar modules and then dynamically merge them; various clusters are shown by different colors. **(C)** Eigenmodules adjacency heatmap. **(D)** The correlation heatmap between the WGCNA module and clinical features. The correlation coefficient and associated p-value are displayed in each column, and positive and negative correlations are denoted by red and blue, respectively. The correlation coefficient increases with color darkness.

To find potential genes linked to AIGVHD, the WGCNA analysis was utilized. The GSE168116 and GSE134662 datasets were integrated, normalized, and the batch effect eliminated (**Supplementary Figure S2**, **Supplementary Table S7**). Based on assessments of scale independence and mean connectivity, a soft power threshold of 9 was determined to be optimal (**Figure 2A**). Following the merging of co-expression modules, 13 separate modules were found, each of which was symbolized by a different color. (**Figures 2B, C**). Notably, the blue and black module was identified as the primary module of interest (**Figure 2D**). Consequently, a total of 140 genes were classified as candidate genes for further investigation based on criteria of |MM| ≥ 0.8 and |GS| > 0.4 (**Supplementary Table S8**).

### 3.3 Identify critical genes and assess diagnostic efficacy

**Figure 3:**
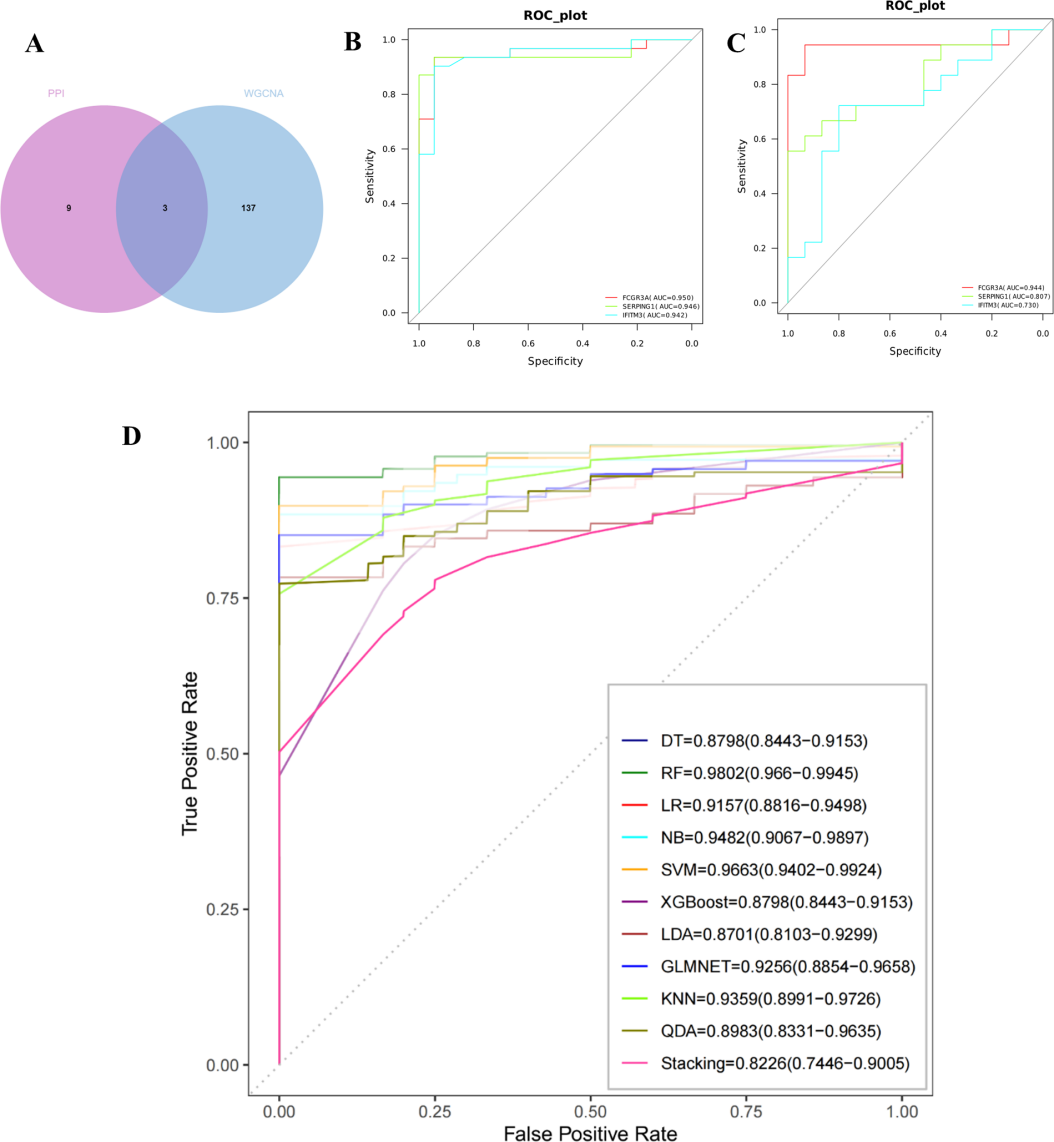
Evaluation and validation of critical genes and the construction of machine learning models. **(A)** Use a Venn diagram to represent the intersection of candidate genes from the PPI network and the WGCNA analysis. Obtain three key genes: IFITM3, SERPING1, and FCGR3A. **(B)** The ROC curves of the three critical genes in the training cohort. **(C)** The ROC curves of the three critical genes in the validation cohort. **(D)** The ROC curves of the 11 machine learning models, which were built using three genes (IFITM3, SERPING1, and FCGR3A).

FCGR3A, SERPING1, and IFITM3 were identified as critical genes due to their overlap in PPI and WGCNA (**Figure 3A**). The predictive capabilities of these genes were assessed through ROC curve analysis. The results indicated that each of the three genes—FCGR3A (AUC = 0.95), SERPING1 (AUC = 0.946), and IFITM3 (AUC = 0.942)—was highly predictive (**Figure 3B**). Furthermore, the performance of these genes was corroborated in an external validation cohort (GSE215068); AUC values were FCGR3A 0.944, SRPING1 0.807, and IFITM3 0.73 resectively (**Figure 3C**). Collectively, these findings validate the potential of FCGR3A, SERPING1, and IFITM3 as effective biomarkers for the diagnosis of AIGVHD.

### 3.4 Model Performance

As illustrated in **Figure 3D**, eleven machine learning models were developed, all demonstrating robust performance metrics. The models achieved the following AUC values: DT [0.8798 (95% CI 0.8443−0.9153)] 、LR [0.9157(95%CI 0.8816−0.9498)] 、NB [0.9482 (95%CI 0.9067−0.9897)] 、SVM [0.9663(95%CI 0.9402−0.9924)] 、XGBoost [0.8798(95%CI 0.8443−0.9153)] 、LDA [0.8701(95%CI 0.8103−0.9299)] 、LMNET [0.9256(95%CI 0.8854−0.9658)] 、KNN [0.9359(95%CI 0.8991−0.9726)] 、QDA [0.8983(95%CI 0.8331−0.9635)] 、Stacking [0.8226(95%CI 0.7446−0.9005)] and RF [0.9802 (95%CI 0.966−0.9945)]. Notably, the Random Forest model displayed the highest performance among the tested algorithms.

### 3.5 Immune microenvironment in AIGVHD

**Figure 4:**
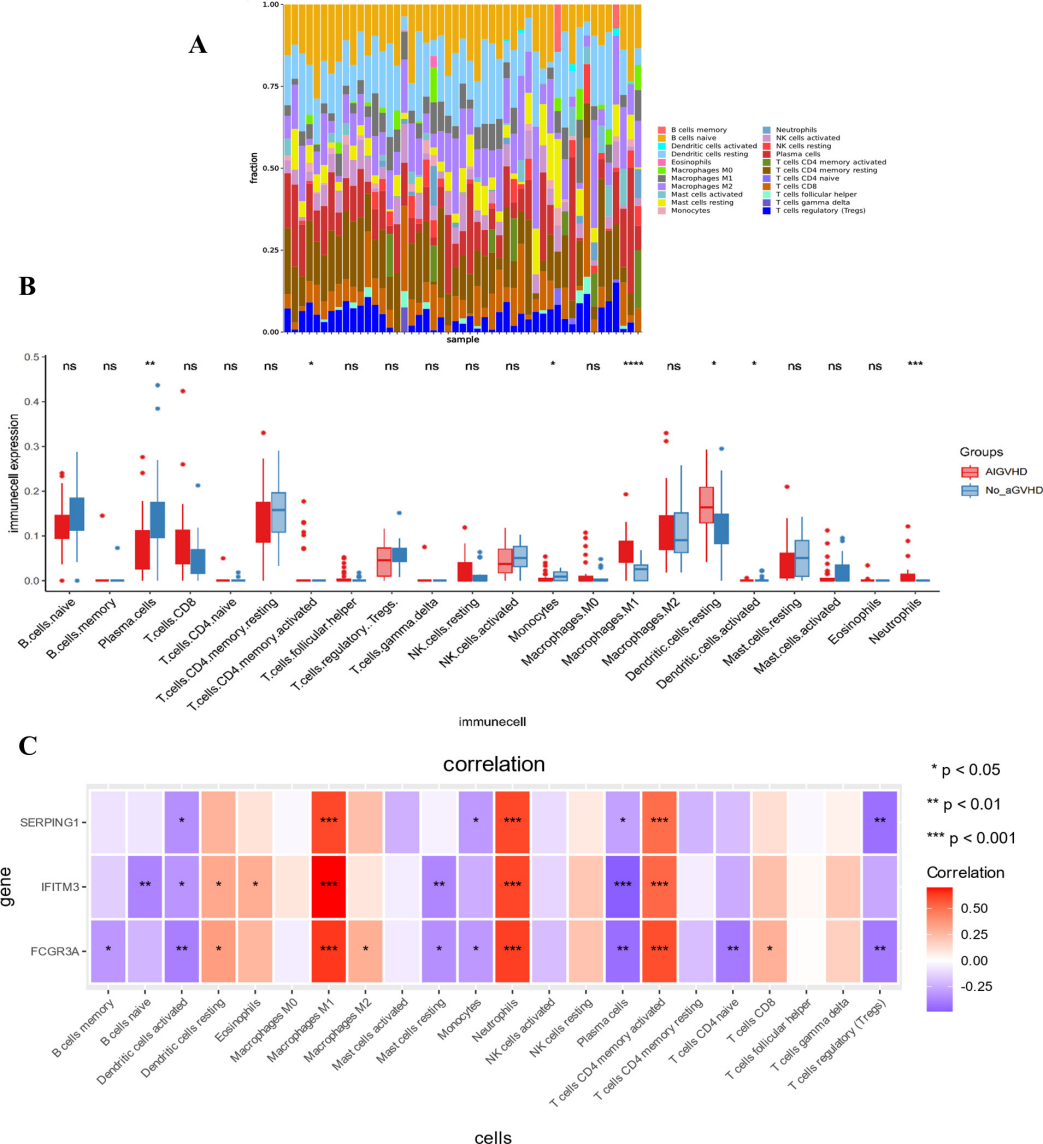
The landscape of immune infiltration and its relationship to critical genes. **(A)** The stacking chart depicts the relative proportion of 22 types of immune cells in each sample. **(B)** The boxplot for expression levels of immune cells between AIGVHD and control group samples. **(C)** The heatmap depicts the relationship between three critical genes and immune cells. *P<0.05, **P<0.01, ***P<0.001, ****P<0.0001. ns, no significant.

A stacked histogram was employed to illustrate the relative proportions of 22 distinct immune cell types, as depicted in **Figure 4A**. Notably, the proportions of plasma cells, monocytes, and activated dendritic cells were significantly lower in cases of AIGVHD, whereas the percentages of M1 macrophages, neutrophils, resetting dendritic cells, and activated CD4 memory T cells were elevated compared to the control group (**Figure 4B**). Furthermore, the gene-immune cell correlation heat map demonstrated that FCGR3A, SERPING1, and IFITM3 exhibited negative correlations with plasma cells, regulatory T cells (Tregs), and activated dendritic cells, while displaying positive correlations with M1 macrophages, neutrophils, and activated CD4 memory T cells (**Figure 4C**).

### 3.6 General condition and aGVHD score

**Figure 5:**
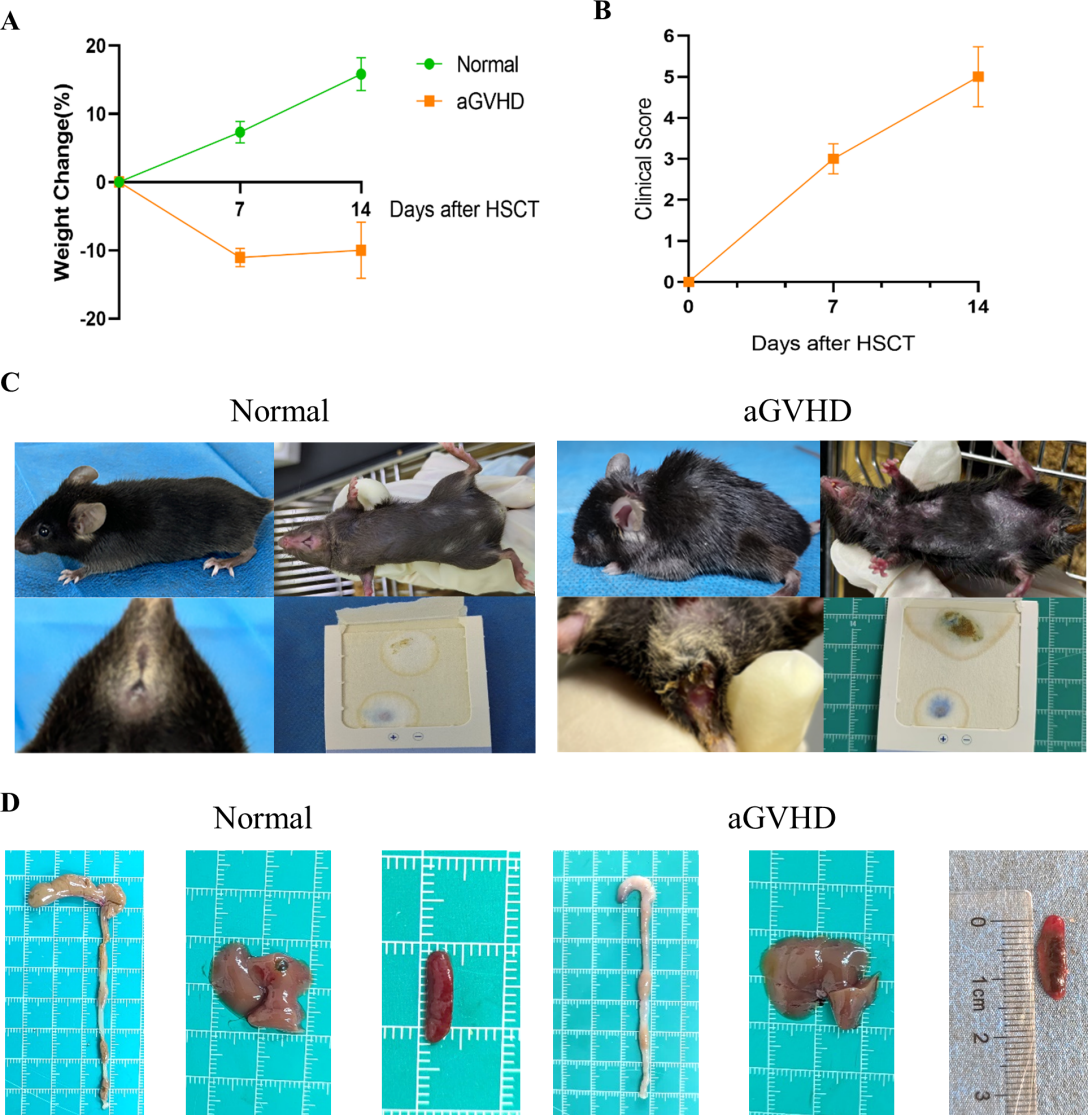
General Condition and Clinical Scoring. **(A)** Weight changes were monitored between the two groups. **(B)** The clinical scoring for aGVHD was assessed within the aGVHD group. **(C)** Clinical symptoms observed in the two groups revealed that aGVHD mice exhibited hunched posture, alopecia, and the presence of bloody stools. **(D)** Anatomical images of the colon, liver, and spleen were obtained from both groups. Data are presented as mean ± SEM (n = 6).

Observations indicated that mice in the aGVHD group exhibited hallmark symptoms of aGVHD, including diarrhea, hair loss, weight loss, hunched posture, and reduced activity levels, with these manifestations intensifying over time. At 7 days post-transplantation, the average clinical score was 3 (moderate GVHD), and at 14 days post-transplantation, it was 5 (severe GVHD). In contrast, control mice demonstrated weight gain and remained asymptomatic (**Figures 5A-C**). Notably, the aGVHD group displayed significant pathological changes compared to the normal group, characterized by cecal congestion, edematous thickening and shortening of the intestinal segments, hepatomegaly, and extensive infarcts within the spleens (**Figure 5D**).

### 3.7 Determination of successful construction of aGVHD mouse model

**Figure 6:**
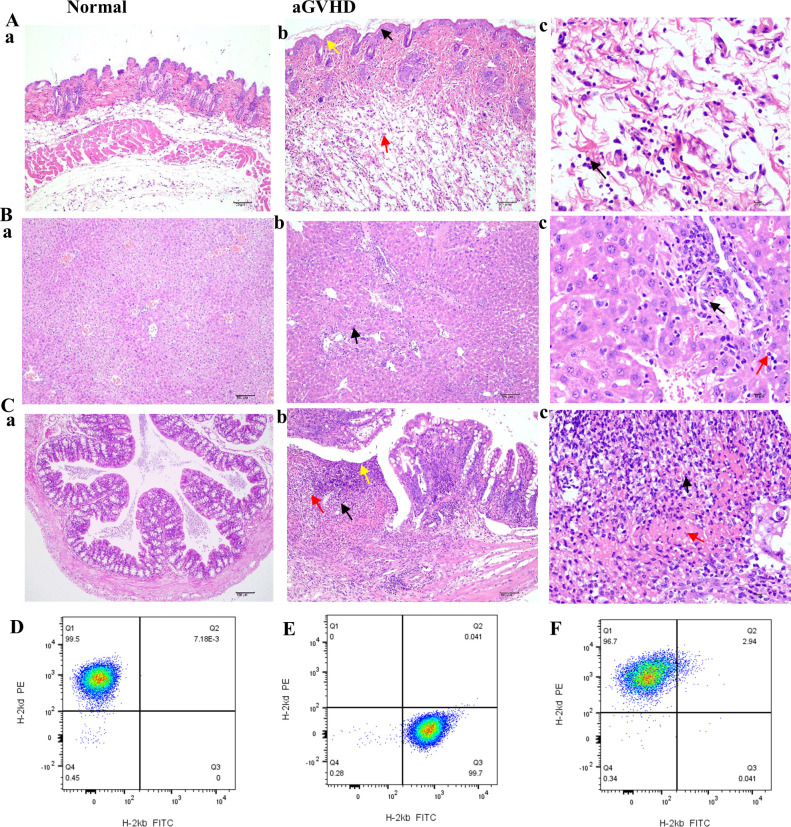
HE staining of GVHD pathological tissues in the skin, liver, and intestine and implantation identification. **(Aa-Ca)** HE staining of skin, liver, and colon from the normal group (×100). **(Ab)** In the GVHD group, a slight thickening of the epidermal layer was noted, accompanied by an increase in the number of spinous layer cells (black arrow, ×100), evidence of mild cellular necrosis (yellow arrow, ×100), and the presence of numerous ruptured adipocytes (red arrow, ×100). **(Ac)** Inflammatory cell infiltration was observed, primarily comprising neutrophils (black arrow, ×400). **(Bb)** In the liver tissue of the GVHD group, minor hepatocyte necrosis was documented (black arrow, ×100); **(Bc)** along with necrosis of a limited number of bile duct epithelial cells (black arrow, ×400). This region exhibited a small degree of inflammatory cell infiltration, predominantly neutrophils with lobulated nuclei (red arrow, ×400). **(Cb)** In the intestinal tissue, the structural integrity of the mucosal epithelial cells was compromised (yellow arrow, ×100), with atrophy or complete loss of intestinal glands (black arrow, ×100) and a notable reduction in the number of goblet cells (red arrow, ×100). **(Cc)** The necrotic region displayed substantial inflammatory cell infiltration (black arrow, ×400) and increased fibrinous exudation (red arrow, ×400). **(D)** BALB/c mice only expressed H-2Kd histocompatibility antigen; **(E)** Only H-2Kb histocompatibility antigen was expressed in C57BL/6J mice before transplantation; **(F)** After transplantation, the GVHD group exhibited expression of the donor histocompatibility antigen H-2Kd.

Compared to the group under control, the skin tissue of the aGVHD group demonstrated significant pathological alterations characterized by epidermal layer thickening, epidermal necrosis, inflammatory cell infiltration, and edema (**Figures 6A**a-c). In the liver, pathological findings included hepatocyte necrosis, inflammatory cell infiltration, and necrosis of biliary epithelial cells (**Figures 6B**a-c). Notably, the colon exhibited a marked deterioration in epithelial structure, a reduction in the population of goblet cells, extensive necrosis of the lamina propria, and pronounced inflammatory cell infiltration (**Figures 6C**a-c). Among the tissues examined, the colonic lesions were the most pronounced in this aGVHD mouse model. These pathological findings align with the Lerner score classification of GVHD, corresponding to grade II-III changes.

The expression of histocompatibility antigens was assessed using flow cytometry. Balb/c mice served as donors, exhibiting exclusive expression of the H-2Kd antigen (**Figure 6D**). Conversely, C57BL/6J mice functioned as recipients, expressing only H-2Kb (**Figure 6E**). However, post-transplantation, in the aGVHD group, there was a marked increase in the expression of the donor histocompatibility antigen H-2Kd, exceeding 95%, thereby confirming successful engraftment of donor cells by day +14 (**Figure 6F**).

**Figure 7:**
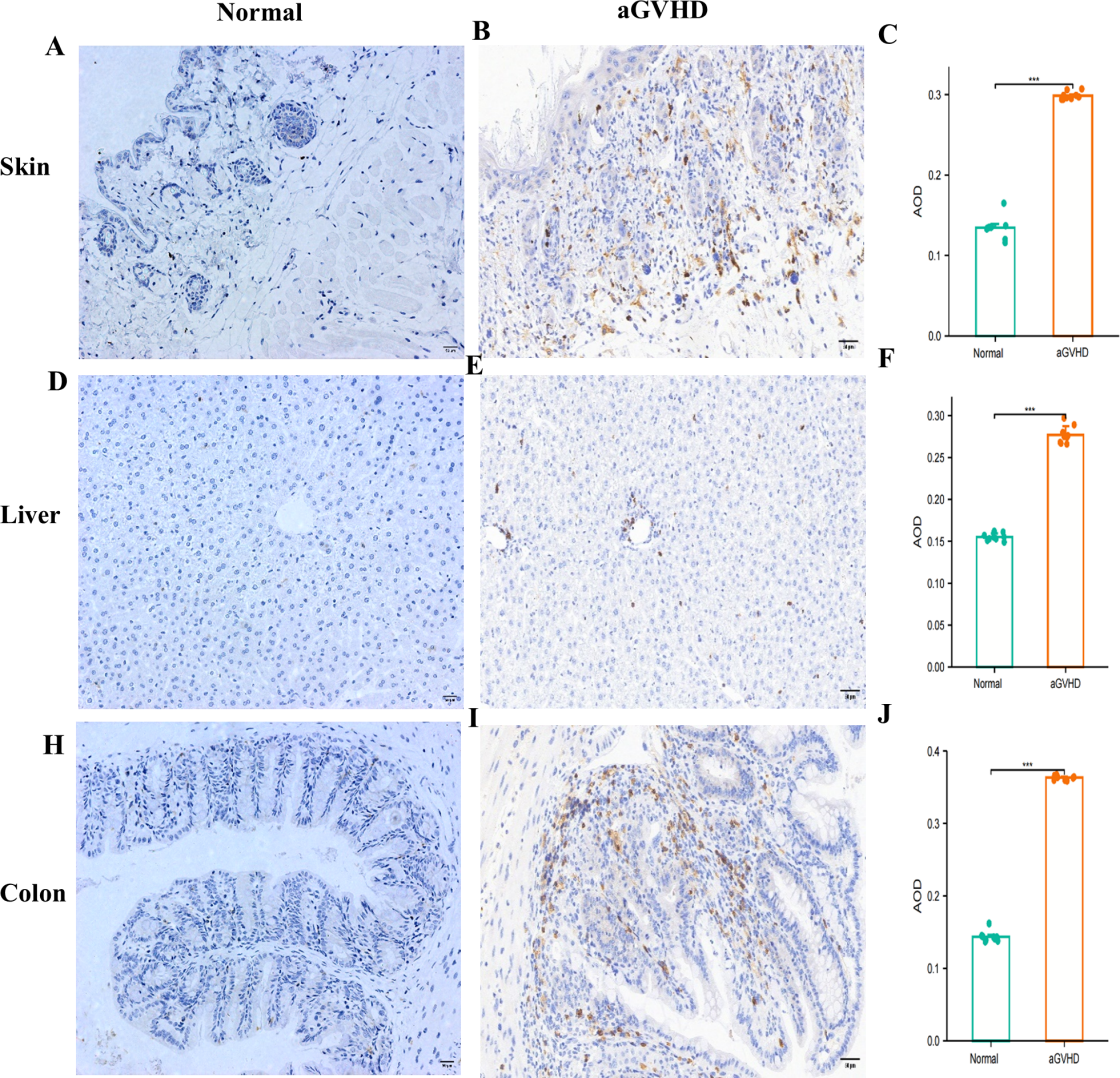
CD4 Expression Analysis.This figure presents a comparative analysis of CD4 expression between the normal group and the aGVHD mouse model in skin tissue **(A-C)**, liver tissues **(D-F)**, and colon tissues **(F-J)**. ***P<0.001.

CD4 was primarily found in the skin tissue’s dermis, the liver tissue’s portal region, and the colon tissue’s submucosa in the aGVHD mouse model. Furthermore, compared to the control group, the AOD values of CD4 in the liver, skin, and colon tissues were significantly higher (**Figures 7A-J**).

### 3.8 The mRNA expression levels of FCGR3A, SERPING1 and IFITM3

**Figure 8:**
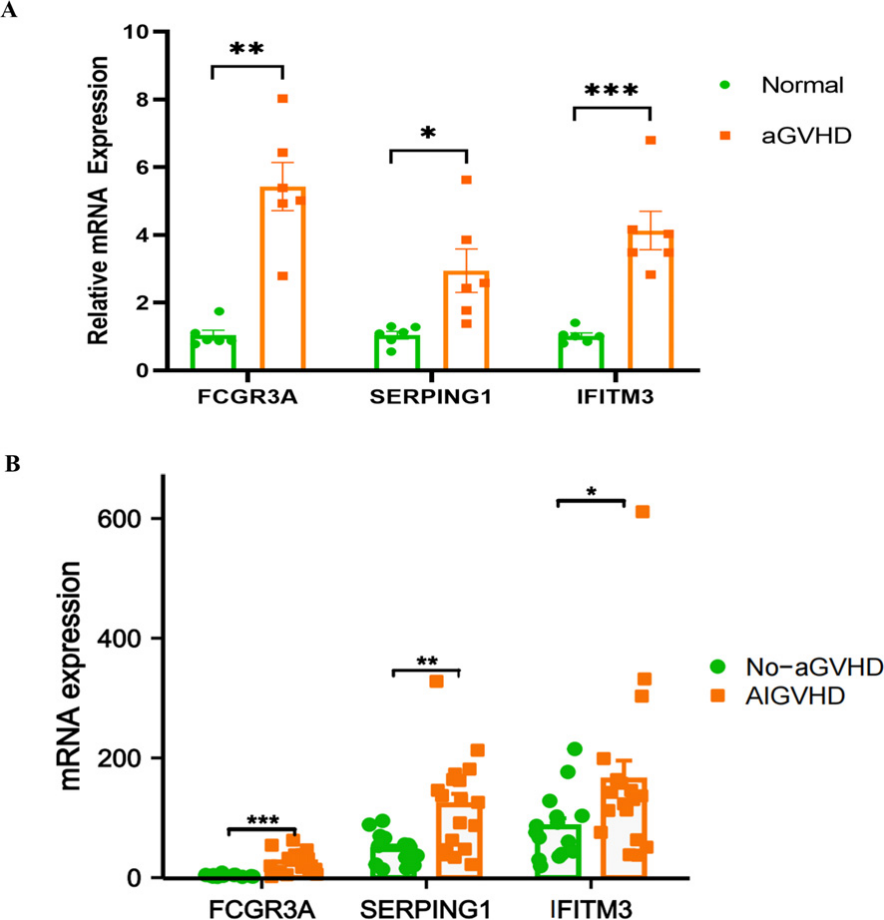
mRNA expression levels of FCGR3A, SERPING1, and IFITM3. **(A)** In murine models, the mRNA expression levels of FCGR3A, SERPING1, and IFITM3 were found to be significantly elevated in the aGVHD group compared to the control group. **(B)** Similarly, analysis of human colon tissues from the GSE215068 series revealed that the mRNA expression levels of FCGR3A, SERPING1, and IFITM3 were markedly higher in samples from patients with aGVHD than in those without GVHD. *P<0.05, **P<0.01, ***P<0.001.

To assess the expression of FCGR3A, SERPING1, and IFITM3 in the intestinal tract of aGVHD mouse models, colons were harvested from the mice 14 days post-transplantation for RT-PCR analysis. The mRNA expression levels of FCGR3A (P = 0.0022), SERPING1 (P = 0.0303), and IFITM3 (P = 0.0003) in the aGVHD group were found to be significantly elevated compared to those in the control group (**Figure 8A**). Through a query of the GEO database, the mRNA expression of the three genes was further validated in human colon tissue samples from the GSE215068 series. The expression levels of FCGR3A, SERPING1, and IFITM3 in samples classified as AIGVHD were significantly elevated compared to those observed in No_aGVHD samples (**Figure 8B**).

## 4 Discussion

AIGVHD is an immune complication with a high fatality rate. Despite recent advancements in research, our comprehension of the underlying pathological mechanisms of AIGVHD remains inadequate, and there is a notable deficiency in specific early diagnostic methodologies alongside effective, individualized treatment strategies. Currently, it is reported that over half of patients suffering from aGVHD demonstrate resistance to first-line glucocorticoid therapy, and a standardized second-line treatment protocol is lacking. Moreover, even with the combination of multiple immunosuppressive agents, clinical outcomes continue to be unsatisfactory (31, 32). Therefore, extensive research into AIGVHD holds significant importance and urgency, particularly for the identification of novel biomarkers that could play a pivotal role in the diagnosis, treatment, and prognosis of this condition.

In order to diagnose AIGVHD earlier and more accurately and to predict prognosis, researchers have focused on investigating biological markers of the disease and creating risk algorithm models in recent years. Studies indicate that patients with GI-GVHD exhibit elevated levels of fecal calprotectin (FC) compared to those with non-GI-GVHD, with levels correlating directly with disease severity (33). The study by Holtan et al. showed that Amphiregulin can predict the severity of aGVHD, treatment response, and patient survival, as well as improve the existing aGVHD risk score system (20). Patel et al. employed spatial transcriptome analysis on gastrointestinal tissues from patients with aGVHD and identified ubiquitin-specific protease 17 (USP17L) as a potential prognostic biomarker for the condition (34). Additionally, Huang et al. reported that elevated mRNA levels of CD25 and CTLA-4 in peripheral blood were linked to gastrointestinal aGVHD, yielding AUC values of 0.6602 and 0.7593, respectively, for the prediction of GI-GVHD (21). Among the most extensively studied plasma biomarkers for gastrointestinal aGVHD are ST2 and REG3 (35, 36). The MAGIC algorithm probability (MAP), derived from these markers, has demonstrated reliability in identifying aGVHD and predicting prognosis (37). Furthermore, Aaron et al. developed a predictive algorithm based on ST2 and REG3, achieving an AUC of 0.75-0.79 for aGVHD diagnosis (15, 38). To date, however, there have been no reports discussing the risk algorithm of tissue-based genetic biomarkers specifically for AIGVHD.

This study employed bioinformatics approaches to identify three critical genes—FCGR3A, SERPING1, and IFITM3—from a transcriptome sequencing dataset derived from the intestinal tissues of patients with aGVHD. Subsequently, ROC curves were used to assess the diagnostic accuracy of these genes for AIGVHD. In the training cohort, the AUC values for FCGR3A, SERPING1, and IFITM3 were 0.950, 0.946, and 0.942, respectively, while the corresponding AUC values in the validation cohort were 0.944, 0.807, and 0.730. These results indicate that all three genes exhibit robust diagnostic capability for AIGVHD in both cohorts. Furthermore, we developed eleven machine learning models incorporating these three genes, revealing that the RF model demonstrates superior diagnostic performance compared to individual gene assessments, accurately detecting AIGVHD with an AUC of 0.9802 (95% CI 0.966-0.9945). The three genes tested in this study had not been previously mentioned as being connected to aGVHD, but their AUCs for diagnosing AIGVHD were significantly higher than those of CD25 and CTLA-4 reported by Huang et al. Furthermore, among the currently published algorithms, the RF model based on the genes FCGR3A, SERPING1, and IFITM3 in this study has the highest diagnostic efficacy in diagnosing AIGVHD.

The pathogenesis of aGVHD is multifaceted, involving not only T cells but also various other immune cell populations. Research has indicated a predominant presence of M1 macrophages in cases of aGVHD, while M2 macrophages are more prevalent in refractory aGVHD and chronic GVHD (39, 40). Additionally, the gut microbial metabolite trimethylamine N-oxide has been demonstrated to exacerbate aGVHD in murine models by promoting macrophage polarization toward the M1 phenotype (41). In contrast, hUC-EVs-ATO has been shown to mitigate the severity of aGVHD by promoting the transition of M1 macrophages to M2-type macrophages (42). In this study, immune infiltration analysis revealed a significant increase in both M1 macrophages and neutrophils within the intestinal tissues of patients with AIGVHD, aligning with prior research findings. Moreover, we identified a positive correlation between the mRNA expression levels of FCGR3A, SERPING1, and IFITM3 and the presence of M1 macrophages and neutrophils. These results suggest potential crosstalk mechanisms between the identified gene biomarkers of intestinal aGVHD and the populations of neutrophils and M1 macrophages; however, further experimental validation is necessary to substantiate these findings.

The SERPING1 gene encodes a highly glycosylated plasma protein that plays a crucial role in the regulation of the complement system, kinin system, and fibrinolytic system. Furthermore, it exhibits anti-inflammatory properties. Research has indicated that in the context of inflammatory bowel disease (IBD), the expression of the SERPING1 gene is upregulated, potentially correlating with the exacerbation of intestinal inflammatory responses (43). FCGR3A functions as an Fc receptor primarily expressed on the surface of immune cells, playing a significant role in the activation and inflammatory response of these cells. Research has demonstrated that MafB can enhance the phagocytic activity of RAW264.7 macrophages by promoting the expression of Fcgr3 (44). Additionally, the V158F polymorphism within the FCGR3A gene has been identified as a potential predictor of outcomes in bone marrow transplantation, with the V/V genotype associated with a reduced risk of both acute and chronic GVHD as well as improved overall survival rate (45). IFITM3 is an interferon-inducible transmembrane protein that plays a pivotal role in adaptive immunity. It enhances the antigen presentation capacity of dendritic cells and influences the overall profile of the cellular immune response (46). Studies have demonstrated that the absence of the IFITM3 gene can lead to exacerbated inflammatory reactions and an increased incidence of tumor development in mice with chemically induced colitis (47). These findings underscore the critical role of IFITM3 in maintaining intestinal immune homeostasis. This study observed that the expression levels of SERPING1, FCGR3A, and IFITM3 genes were significantly upregulated in the intestinal tissue of patients with aGVHD. We hypothesize that these genes may be involved in the pathological processes of aGVHD through their roles in immune regulation and inflammatory response; however, the precise mechanisms warrant further investigation.

We established a murine model of aGVHD to investigate the relationship between the FCGR3A, SERPING1, and IFITM3 genes and AIGVHD, as well as to lay the groundwork for future research. Currently, the primary model for aGVHD involves transplanting donor bone marrow and splenic cells into lethally irradiated allogeneic hosts (48). This approach causes a variety of aGVHD symptoms, including inflammation in the liver, skin, and intestines, significant weight loss, and increased mortality. C57BL/6J and BALB/c mice are commonly used as aGVHD animal models due to their distinct MHC classes I and II, allowing for chimeric analysis after allogeneic hematopoietic cell transplantation (49). Another model of aGVHD widely employed to investigate the mechanisms of T cell dysfunction involves injecting parental (P) donor splenocytes into non-irradiated, immunocompetent adult F1 hosts (P→F1 aGVHD) (50). Furthermore, aGVHD can be induced by transplanting human peripheral blood mononuclear cells (PBMCs) into mice with severe immunocompromised immune systems, such as NSG or irradiated NOD/SCID mice (51). Nowadays, lethal total body irradiation (TBI) is the most common conditioning method used in the aGVHD murine model, with very little cytotoxic medication (52). However, it is essential to note that the majority of patients undergoing allogeneic hematopoietic stem cell transplantation in clinical contexts are typically treated with chemotherapeutic agents.

In this investigation, male BALB/c mice (H-2kd) were utilized as donors, while female C57BL/6J mice (H-2kb) served as recipients. The recipients underwent a regimen of cyclophosphamide paired with Baixiaoan’s myeloablative therapy over a duration of 6 days. On the seventh day, the C57BL/6J mice received a tail vein injection of 0.2 ml cell suspension containing 1 × 10^8 bone marrow cells and 2 × 10^8 spleen cells harvested from BALB/c donors. By the fourteenth day following transplantation, aGVHD was evidenced by average clinical scores reaching five, accompanied by typical pathological changes in the skin, liver, and colon. Flow cytometric analysis confirmed a cell implantation rate exceeding 95%, while immunohistochemical staining indicated a significant upregulation of CD4 expression in the tissues of the skin, liver, and colon. These multifaceted assessments corroborated the successful establishment of the aGVHD mouse model. Furthermore, our findings revealed a marked elevation in the mRNA expression levels of FCGR3A, SERPING1, and IFITM3 in the aGVHD model compared to control subjects. These findings imply a potential association of FCGR3A, SERPING1, and IFITM3 with the pathophysiology of aGVHD, thereby providing a foundation for subsequent investigations.

This study yields valuable insights; however, several limitations warrant consideration. Firstly, the sample size of the GEO dataset utilized herein was relatively small. Secondly, while the genes identified—FCGR3A, SERPING1, and IFITM3—demonstrated considerable diagnostic potential, further validation is essential to confirm their clinical applicability, particularly concerning the machine-learning model developed from these genes. Additionally, the gene biomarkers identified in this research are sourced from intestinal tissue and are relevant for use as biomarkers in intestinal biopsies. Nevertheless, further investigation is needed to assess the expression levels and diagnostic efficacy of FCGR3A, SERPING1, and IFITM3 in peripheral blood samples. Lastly, because intestinal tissues are not readily available in the clinic, we confirmed that FCGR3A, SERPING1, and IFITM3 were associated with AIGVHD in aGVHD mouse models rather than clinical specimens. Future experiments will focus on conducting more personalized correlation analyses involving FCGR3A, SERPING1, and IFITM3.

In conclusion, we employed bioinformatics approaches to elucidate the transcriptomic variations and immune cell infiltration between patients without aGVHD and those with AIGVHD. Our analysis identified three pivotal genes—FCGR3A, SERPING1, and IFITM3—as well as two essential immune cell types: M1 macrophages and neutrophils. Subsequently, we developed machine learning models utilizing these three genes and found that the RF model exhibited a robust predictive capacity for AIGVHD occurrence, achieving an AUC of 0.9802 (95% CI: 0.966–0.9945). Additionally, we successfully established an aGVHD mouse model, confirming that elevated mRNA levels of FCGR3A, SERPING1, and IFITM3 were associated with AIGVHD. Collectively, the findings of this study enhance our understanding of the pathogenesis underlying AIGVHD and suggest potential therapeutic targets for future intervention strategies.

## Data Availability

The datasets presented in this study can be found in online repositories. The names of the repository/repositories and accession number(s) can be found in the article/**Supplementary Material**.
