# Supplementary figures and images for: Identification and validation of tissue-based gene biomarkers for acute intestinal graft-versus-host disease(AIGVHD)
Source: Front Immunol. 2025 May 13;16:1574904. doi: 10.3389/fimmu.2025.1574904 (PMC12106017; doi:10.3389/fimmu.2025.1574904)

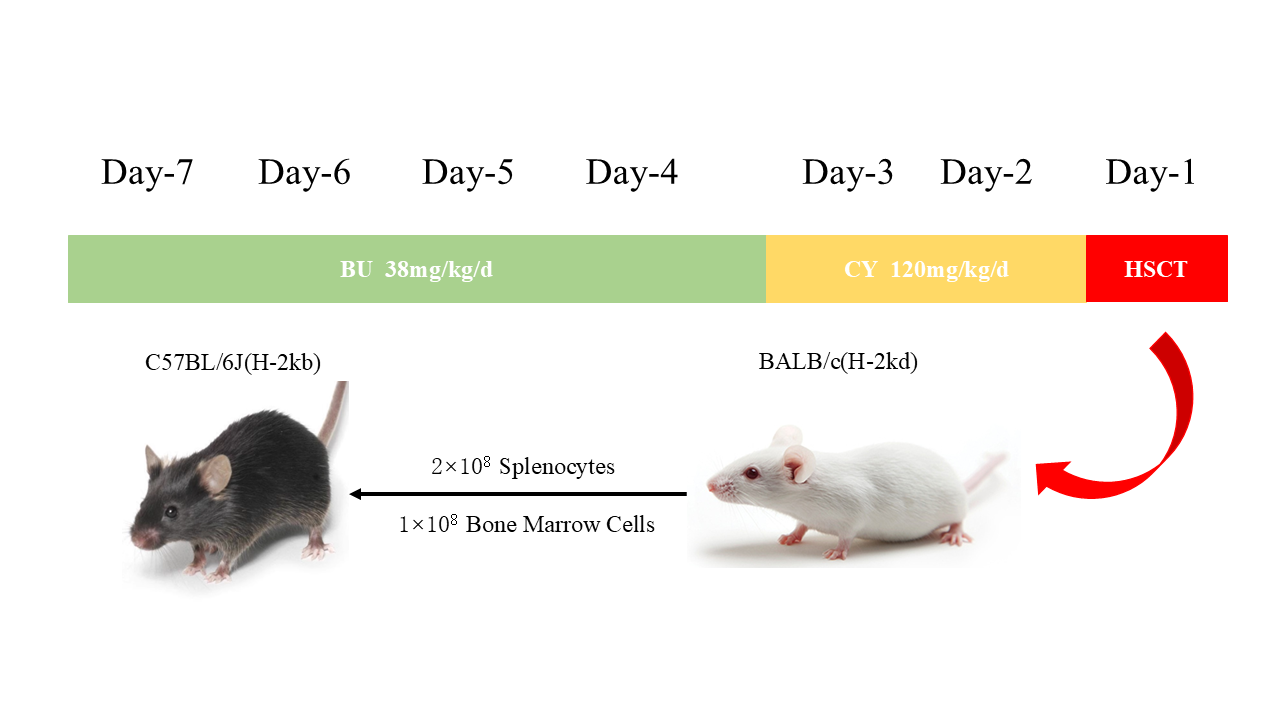

Supplement: Supplementary file 1 [file Image1.tif]

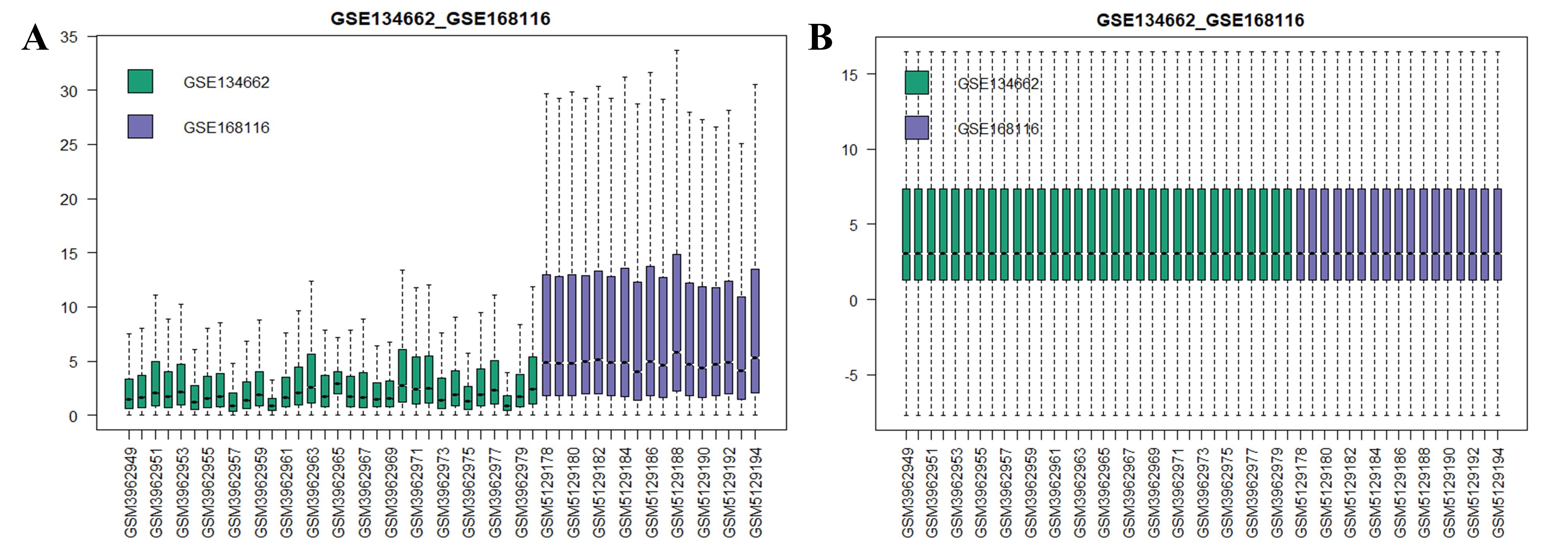

Supplement: Supplementary file 2 [file Image2.tif]
